# Diagnosis of dengue virus infection using spectroscopic images and deep learning

**DOI:** 10.7717/peerj-cs.985

**Published:** 2022-06-01

**Authors:** Mehdi Hassan, Safdar Ali, Muhammad Saleem, Muhammad Sanaullah, Labiba Gillani Fahad, Jin Young Kim, Hani Alquhayz, Syed Fahad Tahir

**Affiliations:** 1 Department of Computer Science, Air University, Islamabad, Pakistan; 2 Department of ICT Convergence System Engineering, Chonnam National University, Gwangju, South Korea; 3 Directorate of National Repository, Islamabad, Pakistan; 4 Agriculture & Biophotonics Division, National Institute of Lasers and Optronics College, Pakistan Institute of Engineering and Applied Sciences (NILOP-C, PIEAS), Lehtrar Road, Nilore, Islamabad, Pakistan; 5 Department of Computer Science, Bahaudian Zakaria University, Multan, Pakistan; 6 Department of Computer Science, National University of Computing and Emerging Sciences, FAST-NUCES, Islamabad, Pakistan; 7 Department of Computer Science and Information, College of Science in Zulfi, Majmaah University, Al-Majmaah, Saudi Arabia

**Keywords:** Dengue, Deep Learning, Raman Spectroscopy, Plasma, Spectra

## Abstract

Dengue virus (DENV) infection is one of the major health issues and a substantial epidemic infectious human disease. More than two billion humans are living in dengue susceptible regions with annual infection mortality rate is about 5%–20%. At initial stages, it is difficult to differentiate dengue virus symptoms with other similar diseases. The main objective of this research is to diagnose dengue virus infection in human blood sera for better treatment and rehabilitation process. A novel and robust approach is proposed based on Raman spectroscopy and deep learning. In this regard, the *ResNet101* deep learning model is modified by exploiting transfer learning (TL) concept on Raman spectroscopic data of human blood sera. Sample size was selected using standard statistical tests. The proposed model is evaluated on 2,000 Raman spectra images in which 1,200 are DENV-infected of human blood sera samples, and 800 are healthy ones. It offers 96.0% accuracy on testing data for DENV infection diagnosis. Moreover, the developed approach demonstrated minimum improvement of 6.0% and 7.0% in terms of AUC and Kappa index respectively over the other state-of-the-art techniques. The developed model offers superior performance to capture minute Raman spectral variations due to the better residual learning capability and generalization ability compared to others deep learning models. The developed model revealed that it might be applied for diagnosis of DENV infection to save precious human lives.

## Introduction

Dengue virus (DENV) infection is a substantial epidemic infectious human disease (Hasan et al., 2016; World Health Organization, 2020). Female mosquitos’ specie *Aedes aegypti* transmitting dengue viral infection through bites (Cucunawangsih & Lugito, 2017). The patient with DENV infection have severe feelings of headache, fatigue, high grade fever associated with rigor and chill (40 °C/104°F), severe retro orbital pain, bone breaking fever, and enlarge petechial rashes (World Health Organization, 2020). DENV infected patient needs special assistance and rehabilitation management plan and if not properly managed, it may cause severe illness and even leads to death. One of the studies reported that there could be 390 million dengue patients worldwide every year. In another study, 3.9 billion persons mostly infected in Asian countries (Brady et al., 2012; Bhatt et al., 2013).

Various laboratory tests are used such as detection of the virus itself (Tsai et al., 2019), antibodies (Narayan et al., 2016) or a combination of both (Chan, How & Ng, 2017). Nonstructural protein-1 (NS1) may be secreted into patients’ blood sera can be used for early DENV infection diagnosis (Lai et al., 2019). After four to five days of illness, virus might be diagnosed using blood serum (World Health Organization, 2009). The clinical tests such as antibodies immunoglobulin M (IgM) or immunoglobulin G (IgG) and IgM/IgG ratios are commonly used for diagnosis of DENV infection (Chatterjee et al., 2016). All these tests are costly and time consuming, therefore, it is inevitable to develop an automated, fast, reliable, and economical DENV diagnostic tests to support the health care system. Owing to inelastic light scattering by molecular vibrations, Raman spectroscopy applied to get the chemical signatures of cells and tissues (Pence & Mahadevan-Jansen, 2016). It has been extensively used for the detection of hepatitis C virus (HCV) (Naseer et al., 2019b), hepatitis B virus (HBV) (Tong et al., 2019; Ali et al., 2021; Saleem et al., 2020), malaria fever (Naseer et al., 2019a) human immunodeficiency virus (HIV) (Lee et al., 2015), tuberculosis (TB) (Stöckel et al., 2017) and DENV infections (Mahmood et al., 2018). For the diagnosis of DENV infection, different multivariate algorithms are employed including principal component analysis (PCA) 20 partial least square regression (PLSR) (Bilal et al., 2017), support vector machine (SVM) (Khan et al., 2016) and random forest algorithms (Khan et al., 2017).

**Figure 1:**
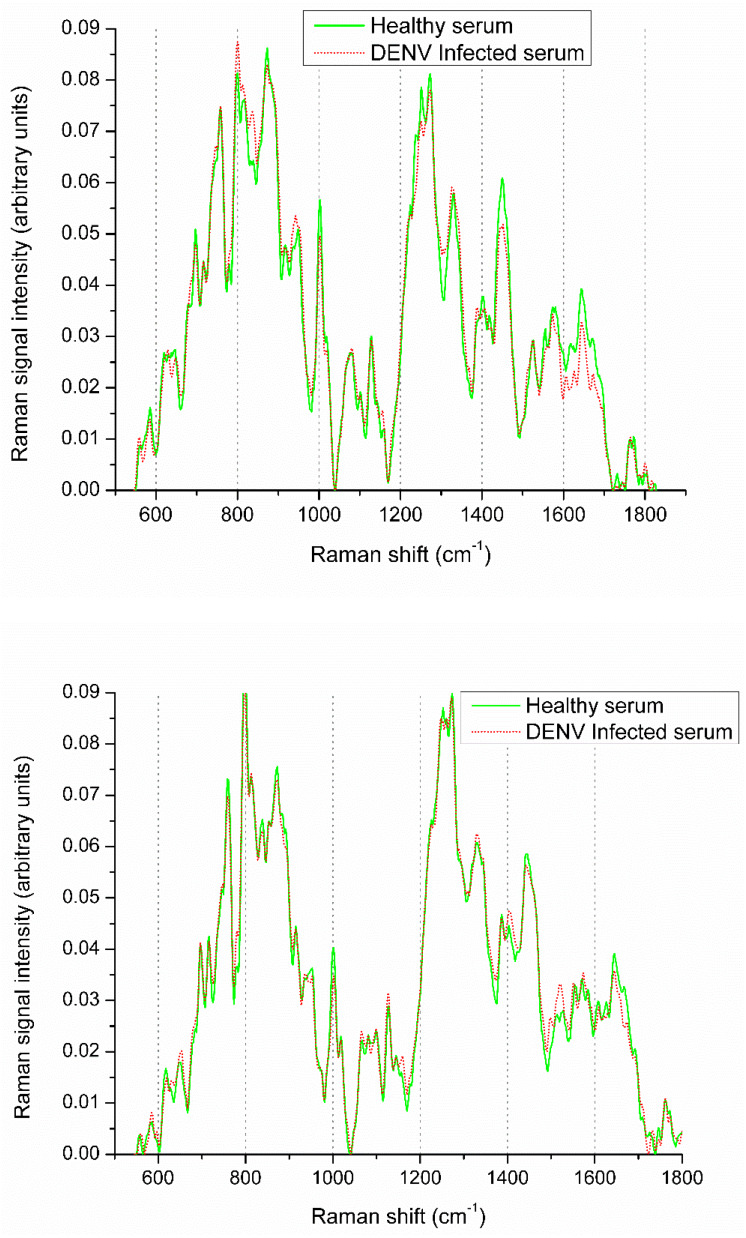
Raman spectra visualization of DENV infected and healthy samples.

Raman spectroscopy is an economical and efficient method of disease diagnosis. However, the diagnosis process involves some challenges; for instance, the variation between healthy and infected spectra is very small (Fig. 1), and it may lead to false diagnosis. Moreover, the conventional machine learning approaches may overlook such minute variations. There is vital to develop a robust and automatic diagnostic technique to differentiate these small spectral variations efficiently. In this paper, we have developed a DEVN infection diagnosis approach using Raman spectroscopic data and convolutional neural networks (CNN) by exploiting the transfer learning (TL) concept. It has an ability to learn the Raman spectra patterns of both infected and healthy samples for accurate diagnosis.

In literature several approaches are proposed by utilizing statistical features and conventional machine learning techniques using Raman spectroscopic data for various disease detection. Koljenović et al. (2007) used Raman spectrum in the range of 2,400–3,800 cm^−1^ to characterize the brain tissues (*ex-vivo* porcine). Kong et al. (2014) varied spectrum range 799–1,098 cm^−1^ for diagnosis of breast cancer. At certain wavelength of Raman spectroscopy, the malignant breast tissues are successfully segregated.

Lin et al. (2018) developed a diagnosis method for *in-vivo* nasopharyngeal cancer by employing reflectance and Raman spectroscopy using fluorescence imaging technique. Orringer et al. (2017) proposed microscopic technique using fiber-laser approach for neuro patients. Similarly, Hollon et al. (2018) used modified fiber-laser imaging for brain tumor detection and reported accuracy between 92–96%. Medyukhina et al. (2012) also detected brain tumor using Raman spectroscopic data. Authors have used image registration techniques for the purpose of tumor boundary estimation, if corresponding mapping varies, it may lead towards misclassification.

Haka et al. (2005) reported breast cancer tissue classification method using Raman spectra. Similarly, Kong et al. (2014) identified breast tumor by applying specific samples of Raman spectra. Magee et al. (2010) explored different Raman spectra for classification of lung tissues into health and diseased. Gao et al. (2012) proposed surgical section tissue estimation using Raman spectroscopy and reported 73% and 74% sensitivity and specificity, respectively. Lieber et al. (2008) proposed skin cancer detection by employing Raman spectroscopy and reported sufficient performance.

Recently, several studies explored machine learning based approaches by employing PCA, LDA, SVM and Random Forest for classification of Raman data. These methods gained popularity among researchers and obtained sufficient performance on Raman spectroscopic data (Smola & Schölkopf, 2004). In summary, these conventional approaches extract various statistical descriptors from Raman spectra data and performed classification tasks.

Khan et al. (2018) used SVM for the detection of HBV from Raman spectroscopic data. They explored various Kernel functions and obtained an accuracy of 98.0%. Amin et al. (2017) used Raman spectroscopic images and proposed DENV infection diagnosis and reported classification performance 96.5% in terms of accuracy by employing PCA-LDA models. Bilal et al. (2016) reported DENV infection classification by employing Raman spectra. In another study, Teh et al. (2008) used Raman spectra for the classification of dysplasia. Ong, Lim & Liu (2012) proposed leukemia cell classification approach and employed PCA.

CNN attracted many researcher across the globe for the solution of real world challenging problems particularly in the domain of medical diagnosis using Raman spectroscopy (Zhang, Ding & Hou, 2020; Dong et al., 2019). Liu et al. (2017) developed chemical species identification model using CNN by employing Raman spectroscopic image data. The reported results show that the CNN based classification model offers better performance compared to PCA and SVM multivariate approaches. Fan et al. (2019), developed CNN models to identify components in a chemical mixture by exploiting Raman spectral features and obtained sufficient performance on both real and simulated Raman spectra as compared to conventional approaches. In another study, Ho et al. (2019) reported classification of pathogenic bacteria by CNN and Raman spectroscopy based approach and yielded 99.70% accuracy. Yan et al. (2020) proposed *“Cornu-Caprae-Hircus”* hydrolysis observation system based on CNN and Raman spectral data. They have developed calibration models by employing live Raman spectroscopy for evaluation of GH hydrolysis using optimized PLS models. Siddiqua, Rahman & Uddin (2021) proposed DNN based approach for the detection of Dengue mosquito. They used various approaches such as faster R-NN, inceptionV3 and ResNet101 in their approach and achieved a good performance.

In general, the variation in healthy and diseased Raman spectra is very small. Such changes occurred due to the small biochemical changes induced by the dengue virus infections. These minute variations make it very challenging for a common clinician to classify diseased and healthy Raman spectra such as DENV infection. To assist this, recent development in machine learning and neural networks have capability to mimic such minute change and classify it efficiently and accurately. In this scenario, we developed a new approach for the identification of DENV infected human blood sera samples using CNN models with TL in combination with Raman images. CNN is efficiently solving the scalability issue and can be applied at enterprise level. This aspect makes our developed models more effective to handle the real time DENV disease identification problem. The new proposed approach based on deep learning and Raman spectra for identification of DENV infected and healthy samples did not explore previously.

The following are main contributions of the proposed approach:

• Statistical study design and sample size selection for validation and trustworthiness of the experiments
• Human blood sera samples of DENV infection and healthy subjects are collected from hospitals
• Raman spectroscopy is employed to obtain the various spectra of healthy and infected samples
• Exploitation of TL concept for improved performance on Raman spectroscopic data of human blood sera
• Design, development, and identification of the best deep learning model TL-ResNet101 among TL-InceptionV3, TL-DenseNet201, TL-GoogleNet, and TL-MobileNetV2 models for DENV infection diagnosis problem
• Modified ResNet101 model by replacing ‘FC_1000’, ‘FC1000_Softmax’ and ‘FC_Classification’ with new layers of ‘FC_2’, ‘FC2_Softmax’ and ‘Class_output’ for present binary class problem
• Built novel framework ‘DENV-TLDNN’ for improved performance of DENV identification
• Automatic approach without any user intervention and no post-processing requirements
• Flexible architecture and can be extended for other types of disease diagnosis

Following are rationales for development of DENV-TLDNN deep learning approach:

• It has residual learning capability which helps the model better classification and generalization capability compared to others deep learning models.
• Secondly, owing to the residual blocks of ResNet101, the vanishing gradient problem is reduced using residual transfers (skip connection). The skip connection concept of ResNet101 model helps to learn an identity function that ensure the better learning performance of succeeding higher residual blocks.

In this way, ResNet101 performs better detection compared to others deep learning models.

The remainder of the article is structured as: Section 2 explains the materials and methods. The experimental results are shown in Section 3; Section 4 describes the discussions. The conclusion and future work are given in Section 5.

## Materials and method

## Materials

### Study design and sample size selection

One of the important steps in the process of designing an empirical study is to select an appropriate sample size for clinical data. If sample size is insufficient or not selected carefully, the study might not be discovered underlying some important treatment effects. An optimal sample size selection saves personnel effort, time, and cost. Sample size must be selected and justified based on statistical calculations. A statistically selected sample size plays crucial role for validation and trustworthiness of the experiments.

**Figure 2:**
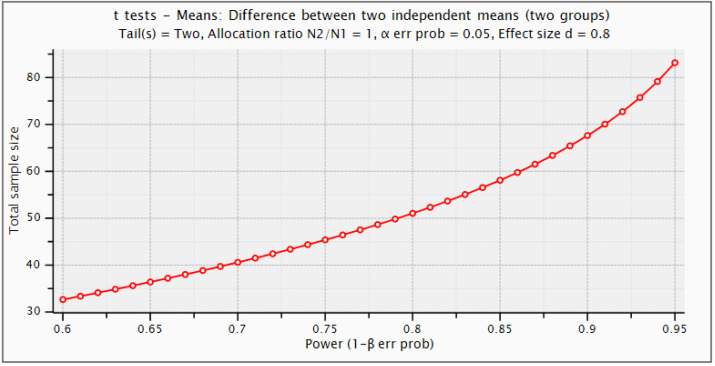
Statistical G*power test for sample size selection.

For this study design, number of samples are determined using power calculations in G*Power 3.1.9.6 (Faul et al., 2007). Statistical power depends on different factors such as effect size, sample size, and the decision criterion (α-value). We used the criteria of 0.20, 0.50, and 0.80 provided by Cohen for small, medium, and large effect sizes, respectively (Cohen, 1992). For this purpose, statistical *t*-test with difference between two independent means are employed. The α error probability (false positive result or type-I error) is set equal to 0.05 and Power (1−β error prob.) that a false negative result or type-II error is considered 0.95. Figure 2 depicts the detectable total sample size as a function of required power (1−β error prob.). Statistical power calculations revealed that this study can be design using minimum sample size of 85 with actual power of 0.952, and minimal effect size of 0.8. However, using this criterion, we have chosen 100 samples (60 Dengue positive and 40 negative samples). The current sample size is sufficient to design this study for DENV infection detection to capture underlying chemical signatures for treatment and rehabilitation of the patients.

### Human blood sera and sample preparation

On the basis of the experimental study design (‘Method’), 60 DENV infected and 40 non-infected blood samples were obtained from different hospitals of Rawalpindi, Pakistan during dengue epidemic outbreak in 2019. Verbel consent of volunteer patients was obtained and approved by the Research and Ethical Committee of Rawalpindi Medical College (RMC) and Allied Hospitals (Holy Family Hospital, Benazir Bhutto Hospital, District Headquarters Hospital, Rawalpindi), Pakistan. Collected samples were kept in freezer and thawed before recording Raman spectra. For acquisition of Raman spectra, there is no need of any special preparation.

### Raman spectra collection

The spectra of sera samples were collected using PeakSeeker Pro-785 Raman system with RSM-785 microscope. The associated diode laser @785 nm was used for acquiring of Raman spectra at resolution of 10 cm^−1^. To focus the laser on sera sample, a 10X objective lens was employed with back-scattering configuration to obtain Raman spectra at the 20 µl quantity placed at aluminum holder. The collected Raman spectra lies in the range of 200–2,000 cm^−1^. Owing to the range 540–1,830 cm^−1^ which contains notable spectral variations is chosen for analysis of spectra.

The presence of additive noise makes it difficult to analyze the recorded spectra at band of interest with naked eyes. Spectral fluorescent of closely bands is visualized and analyzed for annotation. Prior to analysis, it is imperative to reduce the effect of noisy bands in the Raman spectra. All these spectra were pre-processed using Matlab2014a for noise reduction, reference point normalization and correction to make the bands of interest and groups recognized precisely. The polynomial of degree 5 with 13-point window size is used for smoothing by employing Savitzky–Golay method. The fluorescence backgrounds of all spectra were corrected by the removal of baseline.

### Dataset of Raman images

In this study, we have a dataset of 100 subjects (60 infected and 40 non-infected healthy subjects). For each subject, 20 Raman spectra images were acquired to prevent any experimental artifacts, human error, and for an unbiased statistical analysis. These spectra were recorded from different positions by shining a laser power of 150 mW for 10 s. Such 20 different spectra of a subject are reasonably different from others. These images are varying in intensities while keeping the same molecular changes. In terms of intensity variation, all 2,000 Raman spectra are different from each other. Out of 2,000 spectra 1,200 are infected and 800 are healthy (Naseer et al., 2019b; Saleem et al., 2020).

## Method

**Figure 3:**
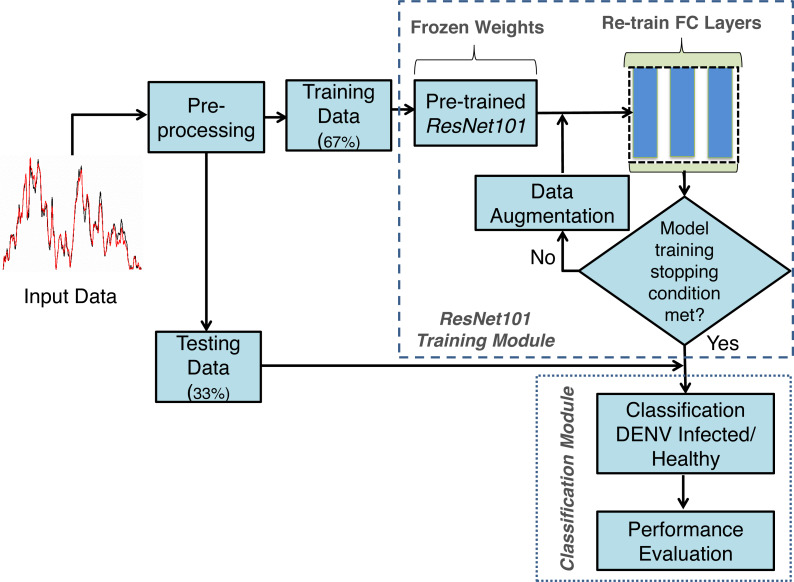
The proposed framework of the DENV-TLDNN.

The proposed framework developed using TL concept on Deep neural networks for DENV infection identification DENV-TLDNN is shown in Fig. 3. It comprised Raman spectra of human blood sera as an input followed by pre-processing and modified *ResNet101* Deep learning model development. For relatively small size of annotated dataset, TL utilizes the power of partially learned model weights to solve the new challenging classification problem efficiently. To validate the effectiveness, DENV-TLDNN is assessed on unseen dataset. Following sub-sections explain components of the proposed approach in detail.

### Pre-processing

The only single pre-processing step is to resize the Raman spectra for compatibility of *ResNet101* input of size 224  × 224 by employing TL concept. The whole dataset was resized and fed it to the modified *ResNet101* for training and testing. The dataset is randomly divided into 67%:33% ratio for model development and testing respectively, as shown in Fig. 3. In first step, modified ResNet101 deep learning model is developed on 67% of dataset. After successful training, the TL-ResNet101 model is evaluated on independent 33% unseen dataset which is not used during model training. All experiments are performed on Intel Xeon E-2246G, 3.6 GHz processor using NVIDIA GeForce RTX-2080 GPU.

### Convolutional neural networks (CNN)

Owing to precision and scalability, CNN gains popularity among researchers. It is being widely used in several real-life applications such as, computer vision, pattern recognition, medical disease diagnosis and natural language processing. CNN has ability to solve complex and non-linear classification problems by extracting most discriminant image features using cascading layers (LeCun et al., 1998). Several convolutional, and pooling layers are stacked to extract most influential features followed by the classification layers.

TL is brilliant idea to use already learned weights of deep neural networks and modify some of the network layers for new classification problems. In such scenario, the modified fully connected layers of the networks are re-train on new dataset. The *ResNet101* deep learning model is trained on ImageNet dataset for 1,000 categories. In this research, the modified *ResNet101* network is trained for binary class instead of 1,000 classes for DENV infection diagnosis using Raman spectroscopic images.

**Figure 4:**
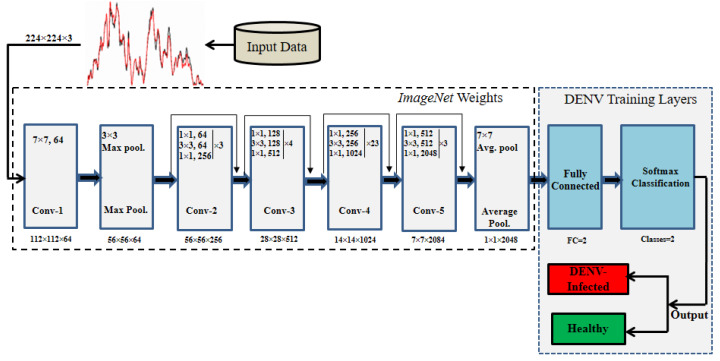
The thematic diagram for *ResNet101-TL* architecture for DENV identification.

To solve the DENV infection diagnosis, *ResNet101* (He et al., 2016) is modified by employing TL concept. DENV infection diagnosis using human blood Raman spectra has not yet explored using Deep TL approach. The thematic diagram of modified *ResNet101* by employing TL concept is shown in Fig. 4. It is highlighted in Fig. 4 that last three layers of *ResNet101* are modified and trained for DENV infection diagnosis. TL and its associated CNN concepts are explained in the following sub-sections.

#### Convolutional layers

Like other CNN, *ResNet101* composed several convolution, pooling, and FC (fully connected) layers which are depicted in Fig. 4. The weights are learned at different stages of convolutional layers using various kernels/filters to generate activation maps for neurons. In this way hidden patterns present in spectroscopic images are explored at various convolutional layers to detect DEVN infection. CNN learns maximum input network response because of its cascading nature of connectivity. The generalization of network is achieved by weight sharing process layer by layer. The output map of the network is obtained using following equations:

(1) \begin{eqnarray*}\begin{array}{@{}l@{}} \displaystyle {M}_{x}^{n}= \frac{{M}_{x}^{n-1}-{K}_{x}^{n}}{{S}_{x}^{n}+1} +1\\ \displaystyle {M}_{y}^{n}= \frac{{M}_{y}^{n-1}-{K}_{y}^{n}}{{S}_{y}^{n}+1} +1 \end{array}\end{eqnarray*}

where, *M*_*x*_ and *M*_*y*_ are the input and output maps respectively with same size maps *M*.*K*_*x*_and*K*_*y*_ represent kernel sizes with *S* and *n* stride rate and layer index, respectively. Additionally, pooling layers are embedded in convolutional layers as shown in Fig. 4.

### Pooling layers

These layers are used to reduce image size by down sampling for translation invariant and decreasing computational cost through dimension reduction. For object identification, max pooling is a well-known operation to be performed (Yamashita et al., 2018; Zhao et al., 2018). The result of max pool *y*_*i*_ is obtained by the following equation:

(2) \begin{eqnarray*}{y}_{i}=\max _{1\leq j\leq M\times M} \left( {x}_{j} \right) ,{x}_{j}\in {\mathbf{X}}_{i}\end{eqnarray*}

where $\mathbf{X}= \left\{ {\mathbf{X}}_{0},{\mathbf{X}}_{1},..,{\mathbf{X}}_{n} \right\} $ combines local areas **X**_0_, **X**_1_, .., **X**_*n*_ of input image. For instance, for *i* ^*th*^ sub-image ${\mathbf{X}}_{i}= \left( {x}_{1},{x}_{2},..,{x}_{M\times M} \right) $ each element of *x* have *M*size. The pooling operation identifies the discriminant image features used DENV identification from Raman spectra. We used a kernel size 2  ×  2 of stride *S* = 2.

### Cost function

It assesses the difference between target and networks predicted output in feed-forward fashion. Cross entropy is utilized as cost function for deep neural networks training. Generally, multi class cost function is defined in Eq. (3).

(3) \begin{eqnarray*}LF=- \frac{1}{N} \sum _{i=1}^{N}{z}_{i}.\log \nolimits \left( p \left( {z}_{i} \right) \right) + \left( 1-{z}_{i} \right) .\log \nolimits \left( 1-p \left( {z}_{i} \right) \right) \end{eqnarray*}

For our binary classification problem, Eq. (3) is modified as follows:

(4) \begin{eqnarray*}L{F}_{Binary}=-{z}_{i}.\log \nolimits \left( p \left( {z}_{i} \right) \right) + \left( 1-{z}_{i} \right) .\log \nolimits \left( 1-p \left( {z}_{i} \right) \right) \end{eqnarray*}

where, *z* and $p \left( z \right) $are output class labels and corresponding probability.

### FC layers

The image features obtained from the ‘*avg_pool’* layer of *ResNet101* are fed to the FC layers to yield output. FC layers produce likelihood for each class generated by the output neurons (classes) which may vary for problem to problem. Finally, softmax function is employed to output neurons (two for our problem) and get predicted class of DENV-infected or non-infected subjects. The softmax function is defined below:

(5) \begin{eqnarray*}soft\text{_}\max \nolimits (\hat {Y}i)= \frac{\exp \nolimits \left( yi \right) }{\sum _{j=1}^{n} \left( yj \right) } .\end{eqnarray*}

### Transfer learning (TL)

Design and development of a deep learning model from scratch required significantly large amount of annotated data. For relatively small dataset, Transfer Learning (TL) is an alternative and popular idea to exploit the pre-learned weights of deep learning models. In this way, the partial knowledge of previously trained model is utilized to solve multiple new classification problems. Principally, TL based deep learning network is to start with pre-initialized weights from training of other related problem and accordingly required less training time. In TL, the existing architectures are modified, and new layers are added for the solution of new problem. In the proposed DENV infection diagnosis, we modified ResNet101 deep learning model by employing TL concept. Three layers of the existing ResNet101 are removed and new layers are added and trained for the diagnosis of DENV infection.

Recently, TL concept gains popularity among researchers that exploit the networks learned weights and used for entirely new classification problem. In TL, the networks weights are frozen except few of the last layers (Russakovsky et al., 2015; Xie et al., 2015). Generally, development of DNN models from scratch requires enormous amount of annotated data. Inherently, medical images labelled data is very small in amount for Deep learning model development. To cope this, it is suitable to modify existing models and apply TL concept on relatively small dataset. TL is employed successfully to classify colon polyps, thyroid, skin cancer, lung nodule, COVID *etc.* (Esteva et al., 2017; Shin et al., 2016; Nayak et al., 2021).

The original ResNet101 was trained for 1,000 classes and having last three layers of ResNet101 namely ‘FC_1000’, ‘FC1000_Softmax’ and ‘FC_Classification’. We modified ResNet101 by replacing the mentioned layers with new layers of ‘FC_2’, ‘FC2_Softmax’ and ‘Class_output’ for our binary classes ‘Infected’, and ‘Healthy’ as depicted in Fig. 4. The ‘avg_pool’ layer of the TL-ResNet101 having 2,048 neurons connected with succeeding layers of two neurons each representing a class. The replaced layers produced high level abstract pattern representation for effective DENV-infection identification.

**Figure 5:**
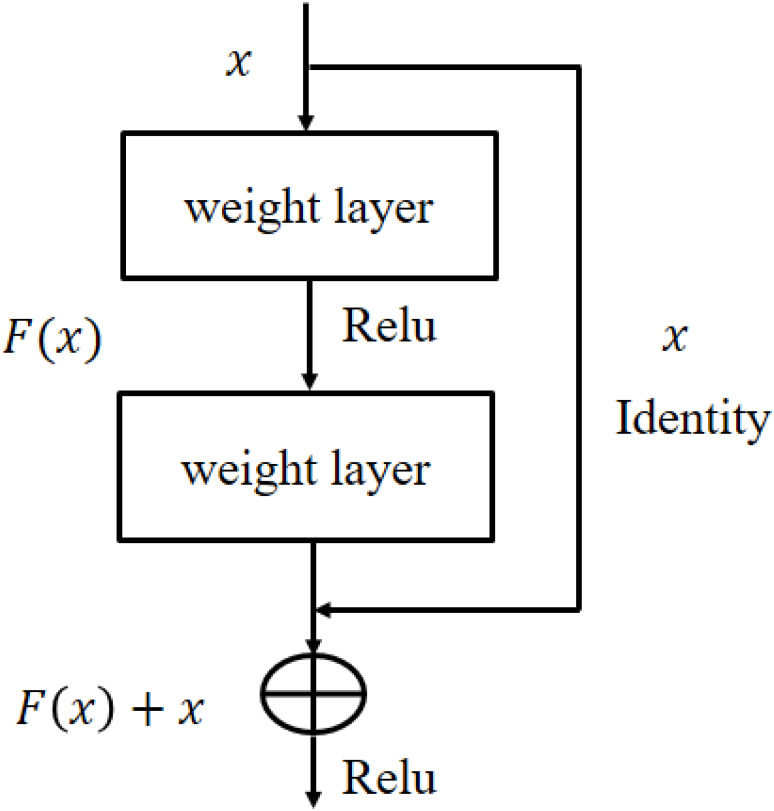
The residual learning flow of ResNet101 architecture.

In training process, hyper-parameters of the networks are tuned for improved performance. The *TL-ResNet101* parameters are set empirically as: learning rate of 0.0001, batch size of 20, data augmentation of (−30, 30) and epochs limit 600 to avoid overfitting and performed generalization. Owing to the residual learning capability and better generalization, *ResNet101* architecture is selected that have advantage over the other architectures such as *AlexNet* and *VGG* (Simonyan & Zisserman, 2014; Krizhevsky, Sutskever & Hinton, 2012). The architectural diagram of residual learning is shown in Fig. 5.

In Fig. 5, *x* and *F*(*x*) are the input and its corresponding activation function. When the activation function produces $F \left( x \right) =0$ the residual process returned an identity *y* = *x*. This process reduces the over-fitting risk hence get better generalization. *ResNet101* architecture detail is given in He et al. (2016).

### Performance assessment

Performance of the developed training and testing models are evaluated using quality measures of accuracy, MCC, F-score ROC (true positive rate *vs* false positive rates), sensitivity, specificity, Kappa index and AUC. These measures are also used to compare the developed approach performance over the other models on the similar dataset. Mathematical definitions of quality measures are given below:

(6) \begin{eqnarray*}Accuracy& = \frac{TP+TN}{P+N} \end{eqnarray*}

(7) \begin{eqnarray*}Senstivity(orrecall)& = \frac{TP}{P} \end{eqnarray*}

(8) \begin{eqnarray*}Specificity& = \frac{TN}{N} \end{eqnarray*}

(9) \begin{eqnarray*}F\text{_}Score& =2\times \frac{precision\times senstivity}{precision+senstivity} \end{eqnarray*}

(10) \begin{eqnarray*}Precision& = \frac{TP}{TP+FP} \end{eqnarray*}

(11) \begin{eqnarray*}MCC& = \frac{TP\times TN-FP\times FN}{\sqrt{ \left( TP+FP \right) \left( TP+FN \right) \left( TN+FP \right) \left( TN+FN \right) }} \end{eqnarray*}

where, P, N FP, FN, and TP represent, total positive, total negative, False Positive, False Negative, and True Positive, respectively.

### Experimental results

The performance of proposed DENV-TLDNN is assessed *via* extensive experiments for diagnosis of DENV infection using Raman Spectroscopic images. The dataset used in this research is obtained from the real patient’s blood samples. The developed model is evaluated by various quality measures and is compared with other state-of-the-art techniques. Results indicated that DENV-TLDNN outperformed over the previously developed approaches. The developed model has potential to use for DENV infection diagnosis at massive scale. Training and testing data randomly split into 67%:33% ratio for model development and evaluation as indicated in Fig. 3. Data splitting presumed that it follows sampling criteria that it marginally distinct the overall data.

**Figure 6:**
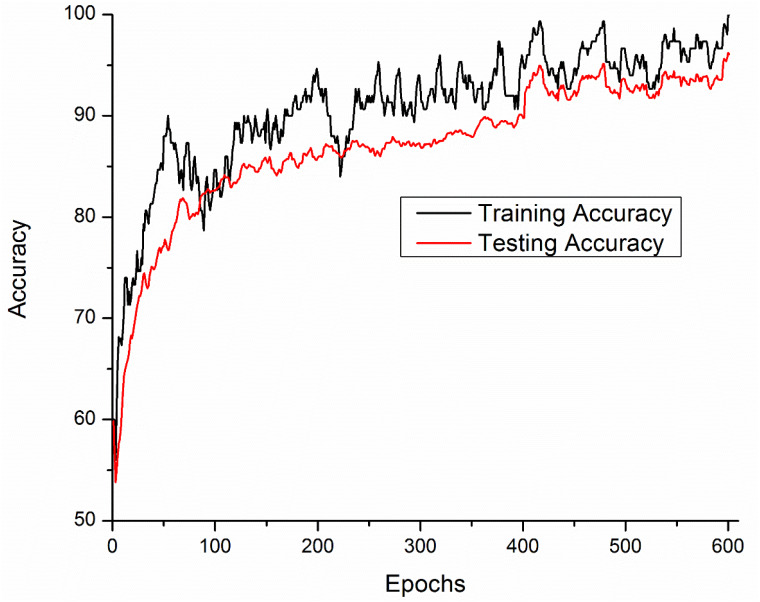
Accuracy performance of the DENV-TLDNN approach.

**Figure 7:**
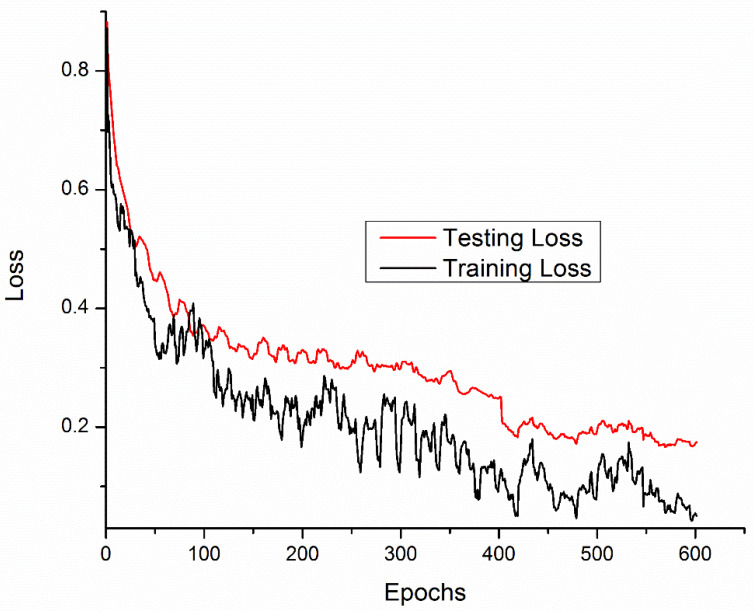
Loss curves for the DENV-TLDNN approach.

**Figure 8:**
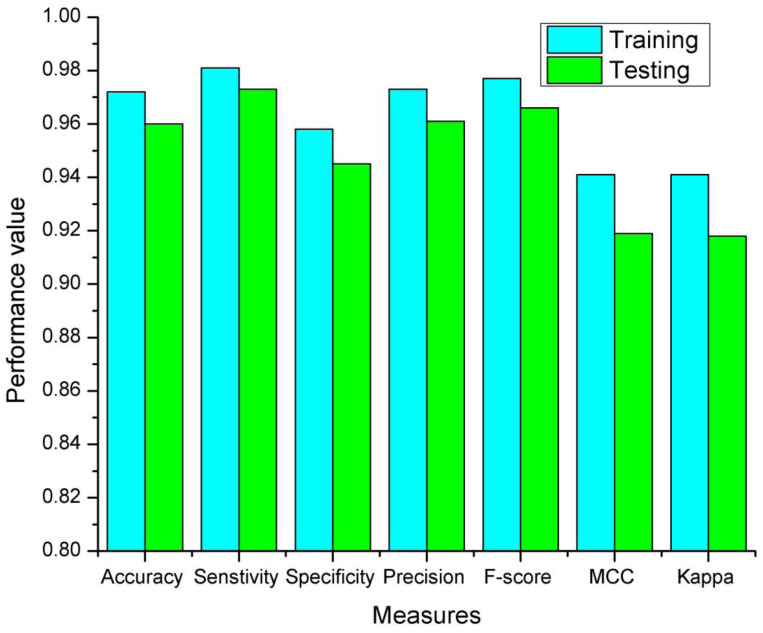
The proposed DENV-TLDNN training and testing measures.

**Figure 9:**
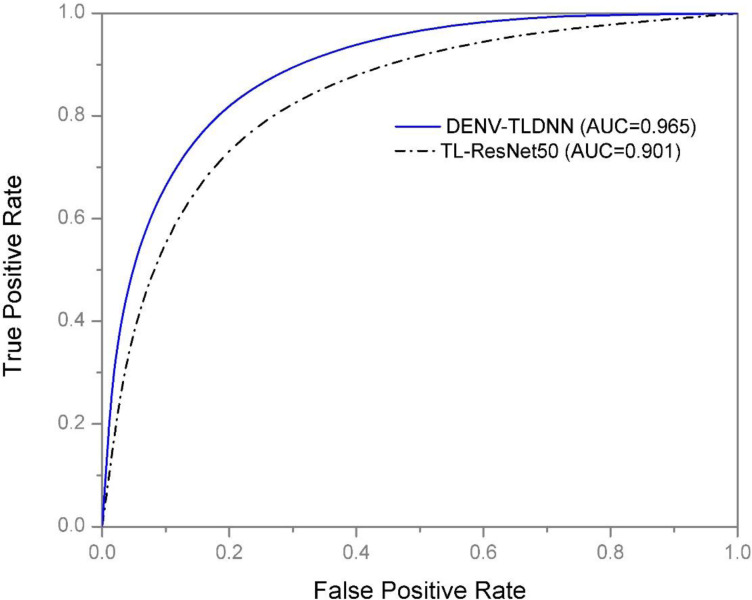
ROC performance of the DENV-TLDNN approach and TL-ResNet50.

Training and testing accuracies along with loss curves of the DENV-TLDNN are shown in Figs. 6 and 7, respectively. Both curves show that model learns parameters consistently and efficiently. Similarly, the average training and testing performance is shown in Fig. 8. Performance comparison in terms of ROC curves of DENV-TLDNN and *TL-ResNet50* is shown in Fig. 9. Moreover, AUC values of the developed and *TL-ResNet50* are 0.965 and 0.901 respectively. Table 1 highlights the various performance comparisons of developed approach with *TL-ResNet50,* and SVM. It is observed from Table 1 that the proposed approach outperformed others. The computational complexity comparison of the DENV-TLDNN and other approaches is shown in Table 2.

## Discussion

Raman spectroscopy provides the opportunity of probing molecular signature of sample and produce qualitative knowledge for decision making. It needs minimal or no sample preparation that made it ideal label free technique for assessing molecular information. Infectious diseases cause a variation in blood chemistry of patients at a certain molecular level with the appearance of antibodies, concentration variance of certain biomolecules in blood sera which can help to disease diagnosis. Deep TL is contemporary approach for robust and efficient diagnosis.

The averaged spectra of DENV-infected and healthy spectra are shown in Fig. 1. It is evident that spectral variations at Raman shifts 622, 647, 680, 698, 746, 760, 800, 814, 840, 873, 890, 944, 1,002, 1,018 and 1,080 cm^−1^ where their relative intensities increase. Whereas, at 1,235, 1,253, 1,273, 1,330, 1,387, 1,405, 1,446, 1,609 and 1,647 cm^−1^ the relative intensities decrease. It is challenging to diagnose DENV-infections using Raman spectra with very small variation present between healthy and infected samples that can be observed from Fig. 1. These spectral variations are exploited to develop the diagnostic model using Deep neural networks.

Raman spectroscopy is an effective and non-invasive and scale invariant for DENV infection diagnosis. It is vital to develop an economical technique to mimic the small variations present in the spectra. The DENV-TLDNN is developed for the accurate diagnosis of DENV infection detection using human blood sera. The blood samples were collected with informed consent of patients.

In this paper, the DENV-infection is diagnosed by Raman spectra data by employing TL concept on *ResNet101* deep learning model. The TL approach is suitable especially when there is relatively small amount of annotated data. For DENV-infection diagnosis problem, last three layers (fully connected, softmax and classification) of the *ResNet101* have been modified. The thematic diagram of TL approach is shown in Fig. 4. It is established that owing to the residual learning (Fig. 5) capability, *ResNet101* offers better generalization as compared to other deep learning models such as AlexNet, VGG and GoogleNet (He et al., 2016).

There are tens of millions of weights which are generated and adjusted during training of deep learning network. These weights are connected through various layers of neurons. Weights of preceding layers acted as inputs to next layers and to an output classification. In this fashion, network learns from very generic to very specific features. Usually, the initial layers of deep learning networks such as ResNet101 model learn very generic features and may also be used a feature extractor. In TL concept, weights of these layers are frozen. Whereas the higher layers of network mimics specific task-oriented features hence used for classification. In TL concept, these layers are removed and replaced with new related problem.

In this study, early layers weights of ResNet101 are frozen (for very generic features) and the higher layers of the network (*e.g.*, ‘FC_1000’, ‘FC1000_Softmax’ and ‘FC_Classification’) are replaced with new layers (*e.g.*, ‘FC_2’, ‘FC2_Softmax’ and ‘Class_output’) for our specific problem of DENV infection diagnosis. In this way, training of newly added layers started to learn specifically weights for DENV infection diagnosis problem. For experimental setup, hyper parameter tuning process has been incorporated and training epochs limit empirically set to 600, learning rate 0.0001, and data augmentation range (−30, 30). To assess networks learning process visually, accuracy and loss functions of the proposed models are shown in Figs. 6 and 7, respectively. It has been observed that the model performance becomes stable after 500 epochs. Although, training curve trends decline, on the other hand, testing error curve start increasing which indicates model sufficiently learn hidden patterns present in the dengue infectious and non-infectious samples. The trained model provides optimal performance at 600 epochs and offers high performance accuracy of 96%. Moreover, it can be observed from Fig. 7 that model testing loss is oscillating even after 500 epochs. This oscillation is due to minute variation present in spectra as observed in Fig. 1. However, at 600 epochs, the variations sufficiently learned and attained 96% performance accuracy.

**Table 1:** Comparison of the proposed approach with other state of the art approaches.

Models	Accuracy	Specificity	Sensitivity	F-Score	Precision	MCC	Kappa
SVM	0.928	0.895	0.950	0.941	0.932	0.850	0.849
TL-ResNet50	0.914	0.889	0.933	0.927	0.922	0.823	0.823
GoogleNet	0.705	0.587	0.864	0.714	0.608	0.457	0.428
InceptionV3	0.736	**1.000**	0.697	0.821	**1.000**	0.482	0.377
MobileNetV2	0.815	0.870	0.794	0.860	0.939	0.613	0.595
DenseNet201	0.826	0.710	0.955	0839	0.747	0.679	0.656
The proposed DENV-TLDNN	**0.960**	0.945	**0.973**	**0.966**	0.961	**0.919**	**0.918**

**Notes.**

The highest values are shown in bold.

**Table 2:** Computational complexity comparison of ResNet101, DENV-TLDNN and SVM.

Classifiers	Classes	Frozen parameters	Parameters at ‘pool5’	Total FLOPS
ResNet101	1000	42606504	2048	44654504
DENV-TLDNN	2	42606504	2048	42610600
SVM	2	–	–	O(n^3^)

For comparison, we have trained ResNet101 and DenseNet201 deep learning model from scratch, *i.e.,* without using ImageNet weights, for DENV infection diagnosis. During the model construction, various data augmentation techniques have been applied to get optimal weights. However, after running 900 and 300 iterations for both models, its performance did not improve and random prediction of models were observed. In medical diagnosis, misclassification have very serious effects. To get high classification performance in the presence of limited dataset, TL might be a good choice.

The modified ResNet101 and DenseNet201 training from scratch offers test accuracy of 39.44% and 55.38% respectively for DENV infection diagnosis. It clearly indicates that these models are not performing well for training from scratch on such limited dataset. Although, we have applied various data augmentation on the data to improve model performance, but it did not work. It is observed that performance of deep learning models (ResNet101 and DenseNet201) trained from scratch offer poor performance over the TL based models.

For relatively small dataset instead of training a deep learning model from scratch, TL is a very popular and highly recommended concept. However, medical diagnosis data is inherently limited. In this scenario, TL is an alternative to solve a new problem. For comparison (Table 1), in addition to modified ResNet101, we have developed other recently deep learning models by modifying InceptionV3, DensNet201, GoogleNet, and MobileNetV2 using TL concept for DENV infection diagnosis problem. Table 1 shows the performance comparison of various approaches for the detection of DENV infection.

For assessment and validation, beside accuracy, some other important classification performance measures such as sensitivity, specificity, precision, F-score, MCC and Kappa indices are given in Table 1. The high values of the classification measures indicated that the DENV-TLDNN can be used for DENV-infection diagnosis. In medical domain, both sensitivity and specificity measures are crucial, and clinicians desires high rate of these measures because treatment and rehabilitation processes are depending on disease detection. Sensitivity of the proposed approach is 97% which represents that detection rate of positive cases is high. On the other hand, specificity of the proposed approach is very high that reduces false positive results. Similarly, all other measures demonstrate the improved model performance.

ROC and AUC are another important performance indicators especially in clinical decision making. ROC shows the diagnostic capability of a model for classification of DENV-infected and non-infected (binary) problem. AUC value of the developed model is 96% which shows good model performance. AUC of developed approach is enhanced by 6% than the *TL-ResNet50* model which indicates its effectiveness compared to others (Table 1). Performance of the DENV-TLDNN in term of AUC and ROC is better compared to *TL-ResNet50* due to: (i) better learning ability of minute Raman spectral variations (ii) use of deeper neural networks and (iii) better generalization capability.

Training and testing results of the DENV-TLDNN are shown in Fig. 8. It is evident from Fig. 8 that training results are better compared to testing and having similar trend to support the validity of robustness model training. This fact validates the effectiveness of the proposed approach.

It is interesting to compare the proposed approach performance with conventional approaches on similar problems. For comparison purpose, we have employed SVM classifier on the DENV infection dataset and it offers 92% classification accuracy as shown in Table 1. The developed DENV-TLDNN approach demonstrated the improved performance over SVM classifier in terms of accuracy, sensitivity, specificity, and Kappa index is 3%, 2%, 5% and 7%, respectively. This enhanced performance shows its efficacy.

Performance comparison of the proposed DENV-TLDNN is compared with other state of the art deep learning models such as, GoogleNet, InceptionV3, MobileNetV2, and DensNet201 by employing TL concept. Table 1 shows the comparison statistics, and it can be observed that the proposed approach offers superior performance at all quality measures. Moreover, the ResNet101 and DenseNet201 models are modified for DENV infection diagnosis and trained from scratch. Various data augmentation steps employed and an accuracy of 39.35% and 55.38% respectively. It is observed that performance of ResNet101 and DenseNet201 which are trained from scratch on relatively small dataset provided poor performance over the TL based models. It is deduced that TL based models satisfactorily improved the performance for DENV infection diagnosis.

For experimental evaluation, computational complexity is one the parameters to assess models’ performance. Usually, the big O notation is used for time complexity calculation of various algorithms. However, in case of CNN models, the computational complexity is assessed in terms of total FLOPS runs on specific hardware. The computational complexity in terms of parameters (FLOPS) of *ResNet101* and the proposed DENV-TLDNN is reduced by 4.6% as shown in Table 2. Whereas the SVM is conventional ML approach with computational complexity **O(n**^**3**^). For testing, both the SVM and CNN models executes in single step. The developed DENV-TLDNN is understandable and reproducible (mathematically and graphically) for verification and evaluation of results.

It is recognized that the developed TL-ResNet101 deep learning models performed well in fact of that: (i) it has residual learning capability and generalization ability compared to others deep learning models; (ii) existence of residual blocks to minimize overcome vanishing gradient problem using skip connection concept. This concept helps to learn an identity function that ensure the better learning performance of succeeding higher residual blocks. In this way, DENV-TLDNN performs better diagnosis compared to other deep learning models.

Generally, training of CNN based approaches are computationally expensive. However, once the final trained model is obtained then its testing does not require much time. Simply, once the model is developed, the practitioners only present the Raman spectra as input and fed to DENV-TLDNN for predication. On the other hand, conventional approaches have several issues, such as pre-processing, feature extraction, filter size, and parameter tuning for classification. However, our developed approach is fully automatic and does not require any user intervention.

In this research, deep TL concept utilized effectively for DENV-infection identification and can be utilized in real time. It is a non-invasive diagnosis approach which did not create discomfort or health hazards to the patients. The only limitation of the developed approach is that it requires a trained staff to acquire Raman spectra and feed it to the DENV-TLDNN for diagnosis.

## Conclusions

In this research, a scalable, reliable, and non-invasive new approach is developed for DENV-infection identification using deep learning and Raman spectra of human blood sera. Deep learning *ResNet101* model is modified by employing TL concept for DENV-infection diagnosis. The proposed approach is evaluated on Raman spectroscopic data of real patients’ blood sera. The developed DENV-TLDNN demonstrated high classification performance at various standard quality measures. The proposed approach offers superior performance over the other state of the art techniques such as TL-ResNet50, TL-DenseNet201, TL-InceptionV3, TL-MobileNetV2, TL-GoogleNet, and SVM. The superior classification performance reveled that the DENV-TLDNN is effective for DENV-infection diagnosis. This work can be extended for other diseases such as tuberculosis, HIV and COVID-19.

## Additional Information and Declarations

### Data Availability

The following information was supplied regarding data availability: The data is available at figshare: Hassan, Mehdi (2022): DENV-TLDNN (Dengue Virus Infection Detection using Deep Transfer Learning and Raman Spectroscopy Data). figshare. Dataset. https://doi.org/10.6084/m9.figshare.18771410.v1.
